# Central and peripheral areal BMD, bone microarchitecture, and strength after initiation of androgen deprivation therapy in men with prostate cancer: a two-year follow-up study

**DOI:** 10.3389/fendo.2026.1862001

**Published:** 2026-08-13

**Authors:** Marsha M. van Oostwaard, Melissa S.A.M. Bevers, Johanna H.M. Driessen, Caroline E. Wyers, Joop P. van den Bergh

**Affiliations:** 1 Department of Internal Medicine, VieCuri Medical Center, Venlo, Netherlands; 2 Department of Rheumatology, Institute of Nutrition and Translational Research In Metabolism (NUTRIM), Maastricht University Medical Center, Maastricht, Netherlands; 3 Department of Biomedical Engineering, Eindhoven University of Technology, Eindhoven, Netherlands; 4 Department of Clinical Pharmacy and Toxicology, Maastricht University Medical Center+, Maastricht, Netherlands; 5 Department of Clinical Pharmacy, Cardiovascular Research Institute Maastricht (CARIM), Maastricht University, Maastricht, Netherlands

**Keywords:** androgen deprivation therapy, bone microarchitecture, bone mineral density, bone strength, dual-energy x-ray absorptiometry, high-resolution peripheral quantitative computed tomography, prostate cancer, zoledronic acid

## Abstract

**Introduction:**

Androgen Deprivation Therapy (ADT) in prostate cancer (PCa) is associated with bone loss and increased fracture risk. Previous studies on the effects of ADT on bone have focused on areal bone mineral density (aBMD) from dual-energy X-ray absorptiometry (DXA) at the central skeleton, while the effects on peripheral aBMD, bone microarchitecture, and bone strength are less well studied.

**Methods:**

In this two-year observational study, we examined the changes in aBMD and volumetric BMD, bone area, microarchitecture, and strength from high-resolution peripheral quantitative CT (HR-pQCT) after ADT initiation in men with PCa.

**Results:**

A total of 115 men were included, of whom 43 were treated with zoledronic acid (ZOL+) from ADT initiation and the other 72 were not (ZOL −), according to national guidelines. DXA (lumbar spine, total hip, femoral neck, total body, left and right arm, left and right leg) and HR-pQCT (distal radius, distal tibia) measurements were performed at ADT initiation (T0) and after 24 months (T2). aBMD in the ZOL− group significantly decreased between T0 and T2 at all measurement locations (*p* < 0.05), with a mean decrease ranging between -3.0 ± 7.4% (left leg) and -5.0 ± 5.1% (femoral neck). In the ZOL+ group, only lumbar spine and right leg aBMD significantly changed (+3.6 ± 4.3% and -2.3 ± 3.2%, respectively). Using HR-pQCT, it was found that bone area, volumetric BMD, microarchitecture, and strength significantly declined at the distal radius and tibia in both the ZOL – and ZOL+ group. The most significant changes were noted at the distal radius in cortical area (ZOL-: -10.1 ± 5.9%; ZOL+: -6.5 ± 5.1%), total BMD (ZOL-: -8.8 ± 4.2%; ZOL+: -5.5 ± 3.7%), and failure load (ZOL-: -11.6 ± 8.0%; ZOL+: -10.2 ± 8.2%).

**Conclusion:**

Two-year ADT in men with PCa led to a significant decrease of central and peripheral aBMD and of HR-pQCT derived bone microstructure and strength at the distal radius and tibia. Although ZOL prevented loss of aBMD in central and peripheral bone, bone area, volumetric BMD, microarchitecture, and strength significantly declined at the distal radius and tibia. These findings emphasize the importance of early risk assessment and guideline-based intervention and highlight the need for continued improvement of bone health strategies in men with prostate cancer undergoing ADT.

## 1 Introduction

Prostate cancer (PCa) is one of the most common malignancies in men and has a high prevalence worldwide (1). Increasing life expectancy and advances in the early detection and treatment of PCa have led to an increase in the number of men living with or having survived PCa (2). Androgen deprivation therapy (ADT) is a cornerstone in the treatment of PCa (3, 4) and has been shown to improve survival (5, 6). However, ADT is associated with significant adverse skeletal effects, including increased bone loss and fracture risk (7–10). While areal bone mineral density (aBMD) starts to decrease in men from the age of 30–40 years by approximately 0.5–1.0% per year, an accelerated annual aBMD loss of 1.5–4% has been reported in men on ADT, particularly within the first 12 months of ADT (11–13). Therefore, current clinical guidelines recommend fracture risk assessment in men starting or undergoing ADT, including measurement of aBMD using dual-energy X-ray absorptiometry (DXA). Based on this assessment, preventive measures, such as anti-osteoporosis medication (AOM), can be prescribed (14, 15).

Most studies on the skeletal effects of ADT have focused on changes in aBMD at central skeletal sites, assessed using DXA. However, DXA cannot discriminate between cortical and trabecular compartments and cannot evaluate bone microarchitecture. To enable assessing cortical and trabecular bone characteristics, peripheral quantitative computed tomography (pQCT) was used in a cross-sectional 12-month study in men with PCa, showing a 7-8% lower BMD at the tibia in men with PCa on ADT than in men with PCa without ADT and in healthy controls (16). High-resolution pQCT (HR-pQCT) enables even more detailed assessment of cortical and trabecular bone, including microarchitecture. These trabecular and cortical microarchitecture properties and strength from HR-pQCT have been reported to predict fractures, independent of aBMD (17, 18). However, the effects of ADT have not been extensively studied using HR-pQCT. In one 12-month observational study of 26 men with PCa, considerable cortical and trabecular microarchitectural deterioration was observed at the distal radius and tibia after the initiation of ADT, as well as a decline in central aBMD (19). A recent longitudinal study by Handfort et al. showed a significant decrease in total and cortical BMD, cortical area, and cortical thickness at the distal radius in men with PCa after 12 months of ADT, as well as a significant decline in aBMD at central sites compared to healthy controls (13). In a follow-up study, the authors reported a reduction in total and trabecular volumetric BMD and mechanical properties at the vertebrae using QCT (20). No significant changes were reported in total body aBMD in that study.

Furthermore, studies on the effects of ADT on aBMD, volumetric BMD, bone microarchitecture, and strength in response to AOM are limited. A randomized placebo-controlled trial (RCT) in men on ADT showed that zoledronic acid (ZOL) administration every three months for twelve months improved aBMD at the hip and spine (21). In another two-year RCT in men on ADT, a single administration of ZOL during ADT improved central aBMD over 24 months, while peripheral (radius) aBMD decreased, while no significant effect on volumetric BMD or bone microarchitecture was reported (22).

These previous studies have provided important initial insights into the effects of ADT bone microarchitecture (in response to AOM). Previous HR-pQCT studies relied on first-generation HR-pQCT, which does not allow direct measurement of all trabecular microarchitecture parameters, and had relatively small sample sizes (n=<100). In addition, follow-up was limited to 12 months in two of the three studies (13, 19), and bone strength was only computed in one study (13). Furthermore, in the RCT evaluating the effect of ZOL using HR-pQCT, ZOL was administered once rather than annually over 24 months. These considerations support the need for further research using updated imaging data and longer follow-up periods. Therefore, the objective of this two-year observational study was to examine changes in central and peripheral aBMD using DXA and in volumetric BMD, bone microarchitecture, and bone strength at the distal radius and tibia using second-generation HR-pQCT in men with PCa on ADT who were or were not treated with annual ZOL according to Dutch guidelines.

## 2 Materials and methods

### 2.1 Study population

This study included data from a multidisciplinary care pathway for men with PCa (PROSTEO-care pathway) implemented as standard care in VieCuri Medical Center, Venlo, the Netherlands (23). The PROSTEO-care pathway aims to prevent bone loss and fractures in men with PCa who are treated with ADT. In this pathway, patients undergo a clinical assessment of their bone health at the initiation of ADT, including a questionnaire, DXA and HR-pQCT measurements, laboratory testing (including primary or secondary hyperparathyroidism, chronic kidney disease, and vitamin D deficiency [serum 25-hydroxyvitamin D3 < 30 nmol/l]), and vertebral fracture assessment (VFA) on radiography (X-ray), as part of routine clinical practice, followed by an annual evaluation (24).

Based on the assessment, lifestyle changes are encouraged, and education regarding fracture risk is provided. In accordance with Dutch guidelines on osteoporosis and fracture prevention (25, 26), men with a *T*-score ≤ -2.0 and/or ≥1 VF of grade 2 or 3 additionally receive annual intravenous administration of 5 mg zoledronic acid (ZOL).

For this study, clinical data were extracted from the electronic records, from men with PCa in the PROSTEO pathway who started ADT (T0) between January 2019 and October 2023 and who had a follow-up period of at least two years (T2). Only data from T0 and T2 were analyzed; data after one-year follow-up were not considered in this study. Patients with bone metastases, previous ADT treatment, or >1 administration of ADT prior to entering the pathway, and those with prior or current use of AOM were excluded from the study. The study population consisted of two groups that were analyzed separately: 1) men with PCa treated with ADT and ZOL (ZOL+) according to the Dutch guideline, and 2) men with PCa on ADT who were not treated with ZOL (ZOL−), or any other AOM.

This study was approved by the Independent Medical Ethics Committee of the Academic Hospital Maastricht/University Maastricht (2024-0178).

### 2.2 Data collection

#### 2.2.1 Patient characteristics and clinical data

Data collected at T0 included age, height, weight, and body mass index (BMI). Data on the date of PCa diagnosis (histological confirmation), indication for ADT, and date of first ADT administration were also collected. Ten-year fracture risk for major osteoporotic fracture (MOF) and hip fracture was assessed by the FRAX^®^ tool using the online calculator for the Netherlands (https://www.sheffield.ac.uk/FRAX/tool.aspx?country=30). Questionnaire data on fracture risk factors, previous fractures, medical history, use of glucocorticoids, dietary calcium intake, smoking, alcohol use, and the number of falls and fractures in the previous 12 months were retrospectively extracted from electronic health records. At T2, results from the repeated DXA and HR-pQCT scans were collected. All patients received continuous ADT over the 24-month period with a GnRH agonist (goserelin) and were not treated with intensified hormonal therapy.

#### 2.2.2 DXA measurement

aBMD measurements with DXA (Hologic QDR 4500; Hologic, Bedford, MA, USA) were performed at the total hip (TH), femoral neck (FN), lumbar spine (LS), whole body (WB). Measurements from the left and right arm (LA and RA, respectively), and left and right leg (LL and RL, respectively) were derived from the WB scan. *T*-scores were calculated based on the female (Caucasian) reference database (27) according to the International Society for Clinical Densitometry (ISCD) guidelines (28).

#### 2.2.3 HR-pQCT measurements

Second-generation HR-pQCT (XtremeCT II; Scanco Medical AG, Switzerland) scans were performed at the distal radius and tibia in accordance with the standard protocol (29). Briefly, a 10.2-mm region was scanned using a fixed offset distance at the non-dominant radius and tibia. In case of a prior fracture, surgery, or metal implants at the non-dominant site the contralateral site was scanned. During scan acquisition, the lower arm/leg was immobilized using a motion-restraining holder. When motion artifacts were observed on the preview slice, the scan was repeated once. The quality of the reconstructed scans was assessed using a standard five-point grading system (30). Scans of grades 4–5 were excluded from analysis. From the scans with grade 1-3, the distal radius and tibia were automatically contoured using the software provided with the system; the periosteal contours were manually corrected when visually deviating from the outer bone margins. The contoured bone was evaluated using standard HR-pQCT analyses to quantify total, trabecular, cortical BMD and trabecular and cortical cross-sectional area and microarchitecture (31). Specifically, the quantified parameters included trabecular area (Tb.Ar), cortical area (Ct.Ar), total BMD (Tt.BMD), trabecular BMD (Tb.BMD), cortical BMD (Ct.BMD), trabecular bone volume fraction (Tb. BV/TV), trabecular number (Tb.N), trabecular thickness (Tb.Th), trabecular separation (Tb.Sp), cortical thickness (Ct.Th), and cortical porosity (Ct.Po). For quantification of the microarchitecture parameters, the bone was segmented from the scans by applying a fixed threshold of 320 mg HA/cm^3^ (trabecular bone) and 450 mg HA/cm^3^ (cortical bone) after first filtering the scans with a standard Gaussian filter (sigma = 0.8, support = 1). Changes in these parameters between T0 and T2 were determined from the overlapping region of both scans using slice-matching. Additionally, bone strength was computed from the scans using micro-finite element (µFE) analysis according to the standard methodology for HR-pQCT scans (31, 32). For this analysis, the µFE models were generated by converting each segmented bone voxel to an equally-sized cubic hexahedral µFE-element. Using this approach, the models directly represent the geometry and porous structure of the bone rather than modeling it as a continuum. The bone was modelled as an isotropic linear-elastic material with a uniform Young’s modulus of 8.748 GPa and a Poisson’s ratio of 0.3. An axial compression was then simulated in a linear FE analysis by prescribing a longitudinal strain of 1% to the top surface, while constraining the other two orthogonal directions. The bottom surface was fully constrained in all three orthogonal directions. From the simulation results, bone stiffness was computed as the reaction force of the applied displacement. Bone failure load (FL) was estimated using the failure criterion described by Pistoia et al. (*i.e.* 2% of elements exceeding the critical strain of 0.7%) (32). In contrast to the other HR-pQCT parameters, stiffness and FL were determined from the entire scan (*i.e.* without slice-matching or three-dimensional registration). All image processing and µFE analysis steps were performed using the built-in HR-pQCT software.

### 2.3 Statistical analysis

Baseline characteristics from men with PCa and ADT, treated with ZOL (group ZOL+) and without ZOL treatment (group ZOL−) were compared using Student’s t-tests for normally distributed continuous variables, Mann-Whitney U tests for non-normally distributed continuous variables, and Chi-square tests or Fisher’s exact tests for categorical variables. Normality of the data distribution was assessed using the Shapiro–Wilk test. Data are presented as mean (standard deviation) for normally distributed variables and as median (interquartile range) for non-normally distributed variables. The mean values of the HR-pQCT and DXA parameters at T0 and T2 were calculated for both groups. The two-year absolute change in DXA and HR-pQCT parameters was calculated for each patient by subtracting the value at T0 from that at T2. The percentage change was calculated by dividing the absolute change by the value at T0 multiplied by 100. One-sample t-tests were used to test whether the change in the mean absolute and percentage change was different from 0 in both the ZOL+ and ZOL− groups. Additionally, the statistical significance of the difference in changes in the HR-pQCT and DXA parameters between the ZOL+ and ZOL− groups was evaluated using independent t-tests. Individual changes in DXA parameters and BMD parameters from HR-pQCT were compared with least-significant changes (LSCs) from literature. For changes in aBMD, the LSCs were defined as ≥3.5% for LS and TH and ≥4.5% for LS, WB, LA, RA, LL, and RL (33–35). For changes in HR-pQCT based BMD, LSCs were defined as 4% for total and trabecular BMD and 2% for cortical BMD (36, 37).

Differences between groups in the proportion of participants with changes exceeding the LSC were compared using a chi-square test. Statistical significance was set at P < 0.05 for all analyses. The Benjamini-Hochberg false discovery rate was used to correct for multiple testing (38). All analyses were performed using the IBM Statistical Package for Social Sciences for Macintosh, version 26.0 (IBM SPSS, IBM Corp, Armonk, NY, USA).

## 3 Results

### 3.1 Study participants

**Figure 1:**
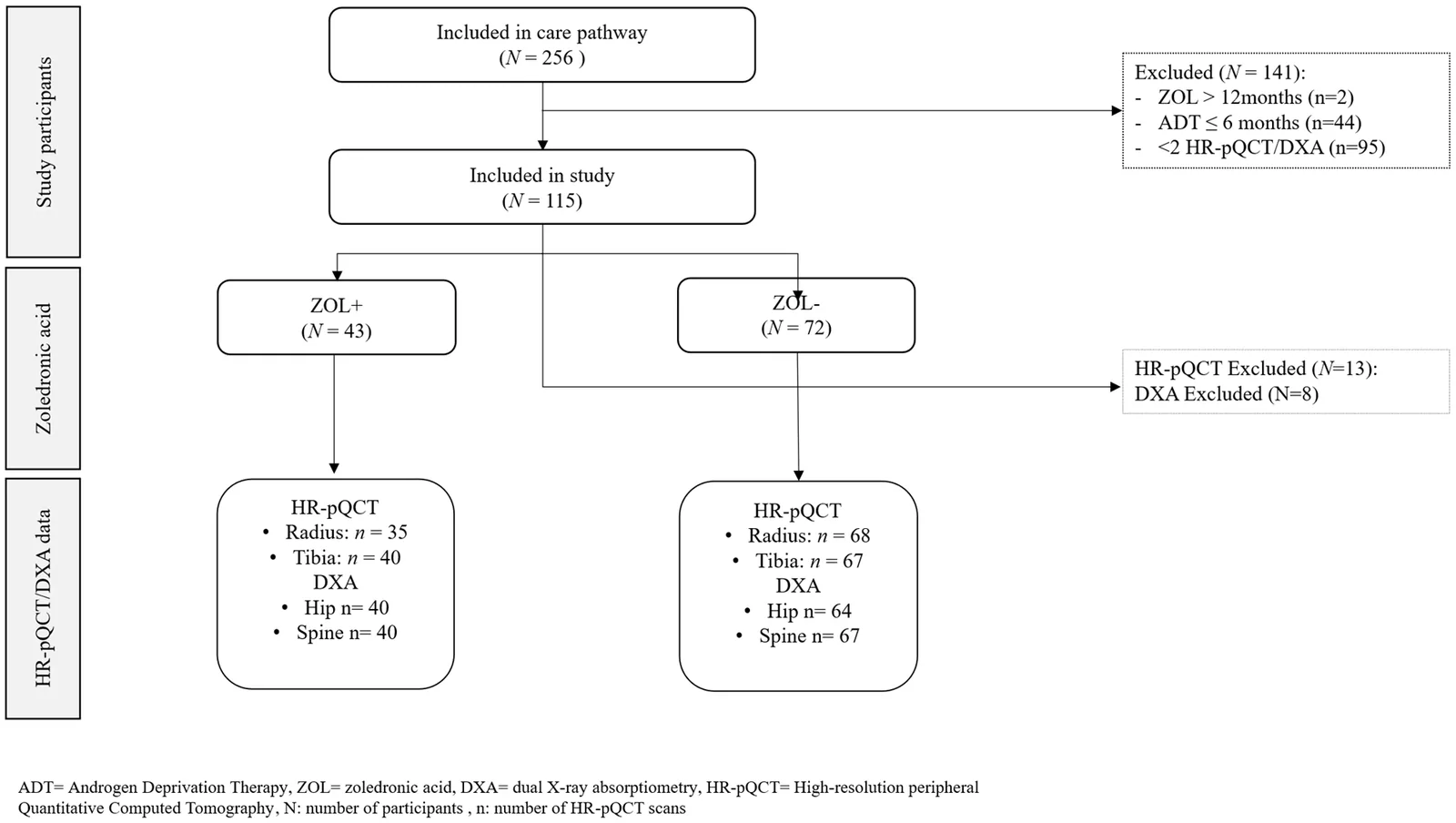
Study design and sample size.

A total of 115 men with PCa who received ADT were included in this study (**Figure 1**), of whom 72 were in the ZOL− group and 43 in the ZOL+ group. Men in the ZOL+ group had significantly higher prostate-specific antigen levels, a higher prevalence of osteoporosis and osteopenia, lower LS aBMD, higher FRAX^®^ scores, and more frequent use of glucocorticoids than those in the ZOL− group. There were no significant differences in age, BMI, Gleason score, FN and TH aBMD between the ZOL- and ZOL+ group (**Table 1**).

**Table 1:** Characteristics of men with prostate cancer at initiation of Androgen Deprivation Therapy (T0) with and without annual zoledronic acid.

	ZOL− (N=72)	ZOL+ (N=43)	P-value
Age (years) [mean, SD]	71.1 (7.6)	73.5 (6.5)	0.065
Weight (kg) [mean, SD]	88.1 (14.5)	81.2 (14.3)	0.747
Height (cm) [mean, SD]	175.9 (7.3)	173.5 (7.1)	0.706
BMI (kg/m^2^) [mean, SD]	28.4 (4.0)	26.9 (4.1)	0.931
Gleason score [no,%] <6 3+4=7 4+3=7 9-10	6 (8.3) 8 (11.1) 6 (8.3) 20 (27.8) 31 (43.1)	3 (7.0) 3 (7.0) 6 (14.0) 16 (37.2) 14 (32.6)	0.577
PSA^1^ [mean, SD]	13.0 (7.60, 27.75)	27.5 (9.87, 63.95)	0.005*
Indication start ADT [no,%] - Primary 2 years (adjuvant) - Long term/continue	61 (84.7) 11 (15.3)	36 (83.7) 7 (16.3)	0.781
Lumbar Spine aBMD (g/cm^2^) [mean, SD]	1.148 (0.161)	1.016 (0.211)	0.041*
Femoral Neck aBMD (g/cm^2^) [mean, SD]	0.820 (0.109)	0.709 (0.114)	0.334
Total Hip aBMD (g/cm^2^) [mean, SD]	1.015 (0.123)	0.894 (0.119)	0.730
Normal aBMD [no, %] Osteopenia [no, %] Osteoporosis [no, %]	59 (81.9) 13 (18.1) 0 (0.0)	12 (27.9) 26 (60.5) 5 (11.6)	0.000*
Indication ZOL [no, %] - T-score ≤ -2.0 - ≥1 vertebral fracture - T-score ≤ -2.0 and ≥1 vertebral fracture	0 0 0	24 (55.8) 15 (34.9) 4 (9.3)	0.000*
Fracture ≥ 50 years [no,%]	7 (9.7)	4 (9.3)	0.922
FRAX -MOF (median, IQR) -Hip fracture (median, IQR)	3.75 (2.70, 4.92) 0.85 (0.50, 1.70)	4.50 (3.30, 7.10) 1.90 (0.90, 3.50)	0.002* 0.000*
Prim hyperparathyroidism [no,%] Vitamin D deficiency [no,%] Chronic kidney Disease [no,%] New known Secondary hyperparathyroidism [no,%] Rheumatoid Arthritis [no,%] COPD [no,%]	1 (1.4) 5 (6.9) 0 6 (8.3) 2 (2.8) 0 8 (11.1)	1 (2.3) 3 (7.0) 1 (2.3) 5 (11.6) 1 (2.3) 0 8 (18.6)	0.710 0.429 0.354 0.883 0.261
Glucocorticoids^2^ [no,%]	1 (1.4)	4 (9.3)	0.044*

Continues variables are described as mean ± SD or median (IQR). Categorical variables are described as number (%).* Statistically significant difference between groups (p < 0.05), PCa, Prostate cancer; ADT, androgen deprivation therapy; BMI, Body mass index; PSA, Prostate-Specific Antigen; FN, Femoral Neck; MOF, Major Osteoporotic fracture; COPD, Chronic Obstructive Pulmonary Disease. ^1^At Prostate Cancer diagnosis ^2^oral/inhaled.

### 3.2 Change in aBMD from DXA

**Figure 2:**
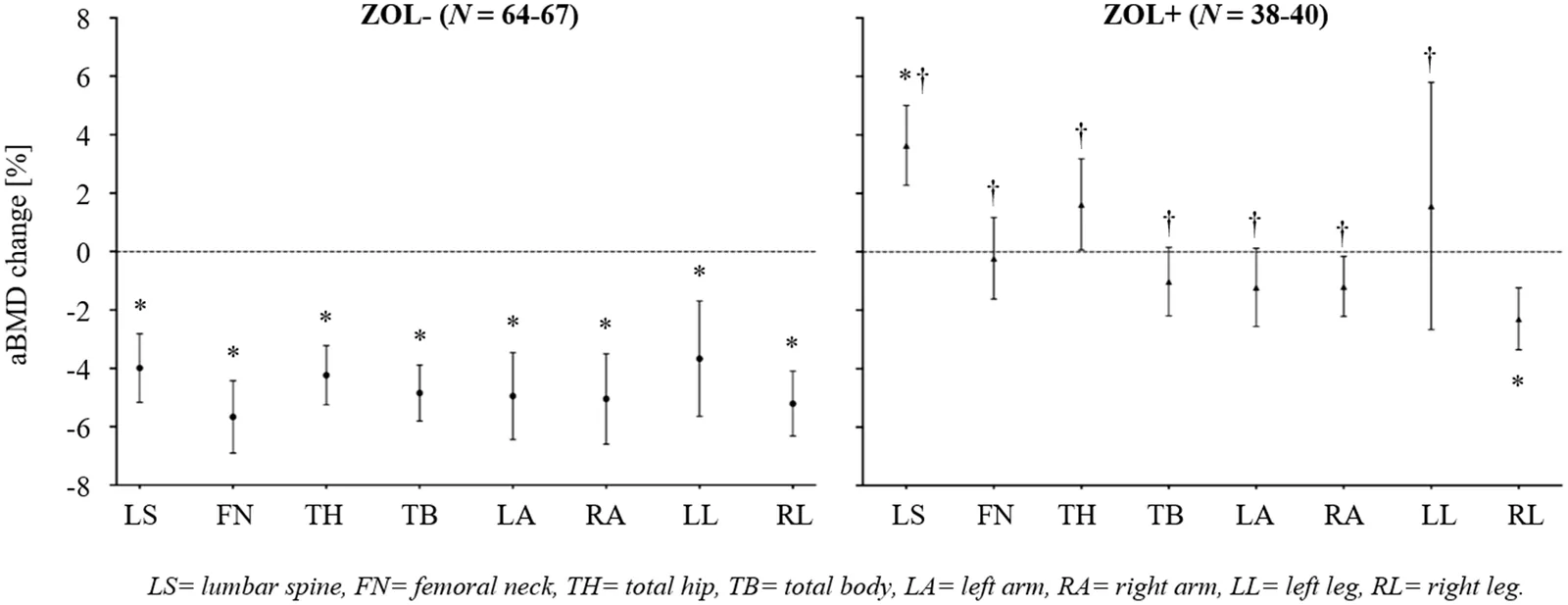
Percentage change in aBMD (g/cm^2^) in men with prostate cancer after two years androgen deprivation therapywithout (ZOL−, left) and with (ZOL+, right) annual zoledronic acid. *significantly different from T0; †significantly different from ZOL−, both after adjustment for multiple testing.

In the ZOL− group, mean aBMD significantly decreased between T0 and T2 at all eight measurement locations (LS, FN, TH, WB, LA, RA, LL, and RL). In the ZOL+ group, mean aBMD significantly increased at the LS and decreased at the RL (**Table 2** and **Figure 2**).

**Table 2:** Longitudinal absolute changes in aBMD (g/cm²) in men with prostate cancer after two years androgen deprivation therapy, with and without annual zoledronic acid.

	ZOL− (N=67)			ZOL+ (N=40)			p-value (absolute)
	Mean T0 (SD)	Mean T2 (SD)	Mean difference	Mean T0 (SD)	Mean T2 (SD)	Mean difference	Δ ADT/ZOL- vs Δ ADT/ZOL+
Lumbar Spine aBMD (g/cm^2^)	1.139 (0.160)	1.103 (0.169)	-0.036 (-0.051, -0.021)**	1.017 (0.216)	1.050 (0.205)	0.034 (0.020, 0.047)**	0.000**
Femoral Neck aBMD (g/cm^2^)	0.816 (0.106)	0.775 (0.108)	-0.041 (-0.052, -0.030)**	0.707 (0.117)	0.705 (0.119)	-0.002 (-0.012, 0.009)	0.000**
Total Hip aBMD (g/cm^2^)	1.009 (0.117)	0.968 (0.126)	-0.041 (-0.052, -0.030)**	0.892 (0.122)	0.905 (0.126)	0.014 (-0.001, 0.028)	0.000**
Total Body aBMD (g/cm^2^)	1.197 (0.120)	1.143 (0.115)	-0.054 (-0.065, -0.043)**	1.115 (0.117)	1.104 (0.121)	-0.012 (-0.026, 0.002)	0.000**
Left Arm aBMD (g/cm^2^)	0.865 (0.069)	0.825 (0.084)	-0.040 (-0.053, -0.028)**	0.810 (0.062)	0.800 (0.066)	-0.010 (-0.021, 0.001)	0.000**
Right Arm aBMD (g/cm^2^)	0.830 (0.072)	0.830 (0.072)	-0.043 (-0.057, -0.030)**	0.819 (0.066)	0.809 (0.066)	-0.010 (-0.019, -0.002)*	0.000**
Left Leg aBMD (g/cm^2^)	1.297 (0.196)	1.258 (0.212)	-0.038 (-0.062, -0.015)**	1.195 (0.159)	1.211 (0.210)	0.017 (-0.033, 0.068)	0.024**
Right Leg aBMD (g/cm^2^)	1.295 (0.183)	1.242 (0.192)	-0.054 (-0.073, -0.034)**	1.234 (0.224)	1.204 (0.204)	-0.031 (-0.049, -0.014)**	0.132

* significant delta (Δ) T0-T2 (p<0.05) ** significant after correction for multiple testing (Benjamini-Hochberg), T0= aBMD measured at initiation of androgen deprivation therapy T2= aBMD measured at 24 months of androgen deprivation therapy; BMD, bone mineral density; DXA, Dual-energy X-ray absorptiometry; ADT, Androgen Deprivation Therapy; ZOL, zoledronic acid 5mg; SD, standard deviation; Missing: ZOL-, Femoral Neck & Total Neck N = 3; Missing: ZOL+, Total Body, arm left, arm right, leg left, leg right N = 2.

When comparing the two treatment groups, the aBMD changes were significantly different in favor of the ZOL+ group (**Supplementary Table 1**).

As shown in **Table 3**, the proportion of men with a decrease in aBMD ≥ LSC at the LS, FN, TH, WB, LA, RA, LL, and RL was significantly higher in the ZOL− group (range between 41.8% (LL) and 59.7% (LA)) than in the ZOL+ group (range between 2.3% (LS) and 18.6% (RA)).

**Table 3:** Proportion of men with aBMD and vBMD decrease > LSC after two years androgen deprivation therapy, with and without annual zoledronic acid.

	ZOL− (n=67)	ZOL+ (n=40)	P-value
DXA			
-Lumbar Spine aBMD (g/cm2)	36 (53.7%)	1 (2.3%)	0.000*
-Femoral Neck aBMD (g/cm2)	33 (49.3%)	5 (11.6%)	0.000*
-Total Hip aBMD (g/cm2)	36 (53.7%)	5 (11.6%)	0.000*
-Total Body aBMD (g/cm2)	29 (43.3%)	6 (14.0%)	0.003*
-Left Arm aBMD (g/cm2)	40 (59.7%)	5 (11.6%)	0.000*
-Right Arm aBMD (g/cm2)	33 (49.3%)	8 (18.6%)	0.003*
-Left Leg aBMD (g/cm2)	28 (41.8%)	5 (11.6%)	0.002*
-Right Leg aBMD (g/cm2)	36 (53.7%)	7 (16.3%)	0.000*
HR-pQCT			
*radius*	(n=67)	(n=35)	
-Total BMD (mg HA/cm^3^)	59 (88.1%)	22 (62.9%)	0.003*
-Trabecular BMD (mg HA/cm^3^)	26 (38.8%)	7 (20.0%)	0.054
-Cortical BMD (mg HA/cm^3^)	58 (86.6%)	29 (82.9%)	0.615
*tibia*	(n=67)	(n=40)	
-Total BMD (mg HA/cm^3^)	56 (83.6%)	24 (60.0%)	0.007*
-Trabecular BMD (mg HA/cm^3^)	5 (7.5%)	0 (0.0%)	0.077
-Cortical BMD (mg HA/cm^3^)	64 (95.5%)	36 (90.0%)	0.264

* Significant (p<0.05).

ADT, Androgen Deprivation Therapy; ZOL, zoledronic acid aBMD, areal Bone Mineral Density; DXA, dual X-ray absorptiometry; HR-pQCT, High-resolution peripheral Quantitative Computed Tomography; LSC, least significant change.

Lumbar spine LSC ≥3.5%, missing n=8, Total hip LSC≥ 3.5%, missing n=8, Femoral Neck Total Body; Arm left; Arm right; Leg left; Leg right aBMD LSC>4.5%, missing n=8.

Tt.BMD= Total body bone mineral density, missing n=13 Tb.BMD=trabecular bone mineral density, missing n=13, Ct.BMD=cortical bone mineral density, missing n=13.

For completeness of the data, we presented the aBMD results at T1 in **Supplementary Table 3**.

### 3.3 Changes in HR-pQCT parameters

**Figure 3:**
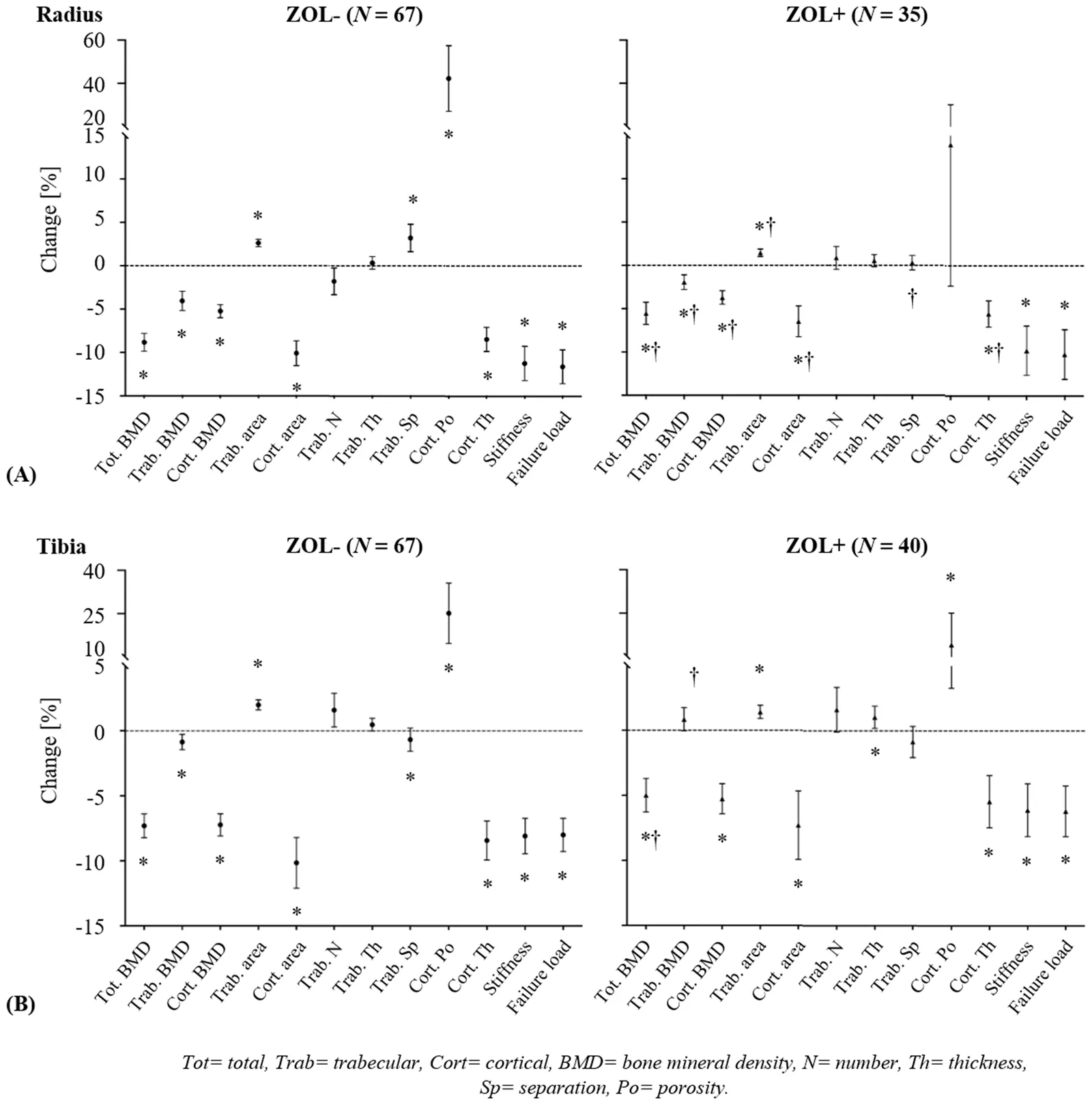
Percentage change in bone area, volumetric bone mineral density and microarchitecture measured with HR-pQCT at the **(A)** distal radius and **(B)** distal tibia.in men with prostate cancer after two years androgen deprivation therapy. The left panels are men without (ZOL−) and the right panels with (ZOL+) annual zoledronic acid. *significantly different from T0; †significantly different from ZOL−, both after adjustment for multiple testing.

In the ZOL− group, Ct.Ar, Tt.BMD, Tb.BMD, Ct.BMD, Ct.Th, stiffness, and FL decreased significantly, and Tb.Ar and Ct.Po increased significantly between T0 and T2, at both the radius and tibia. The trabecular microarchitectural parameters showed less pronounced changes (**Table 4**, **Figure 3**).

**Table 4:** Longitudinal absolute changes in bone area, volumetric bone mineral density and microarchitecture measured by HR-pQCT in men with prostate cancer after two years androgen deprivation therapy, with and without annual zoledronic acid.

	ZOL− (N=67)			ZOL+ (N=35)			p-value (absoute)
	Mean T0 (SD)	Mean T2 (SD)	Mean difference	Mean T0 (SD)	Mean T2 (SD)	Mean difference	Δ ZOL- vs Δ ZOL+
Distal Radius							
*Bone area*							
Tb.Ar (mm^2^)	305.10 (61.78)	312.73 (61.75)	7.63 (6.46, 8.80)**	310.32 (65.11)	314.92 (66.77)	4.61 (3.23, 5.99)**	0.002**
Ct.Ar (mm^2^)	78.64 (16.63)	70.78 (15.88)	-7.86 (-9.01, -6.70)**	72.09 (11.96)	67.42 (11.84)	-4.67 (-6.00, -3.34)**	0.001**
*Volumetric BMD*							
Tt.BMD (mg HA/cm^3^)	320.51 (66.80)	292.67 (65.03)	-27.84 (-31.22, 24.46)**	294.61 (46.99)	278.53 (46.34)	-16.08 (-19.79, -12.38)**	0.000**
Tb.BMD (mg HA/cm^3^)	178.79 (38.94)	172.16 (40.92)	-6.63 (-8.32, -4.94)**	163.17 (29.97)	160.12 (29.85)	-3.05 (-4.48, -1.62)**	0.002**
Ct.BMD (mg HA/cm^3^)	864.58 (53.85)	819.89 (64.60)	-44.68 (-50.91, -38.46)**	853.45 (53.47)	822.13 (58.82)	-31.32 (-37.88, -24.76)**	0.004**
*Microarchitecture*							
Tb.BV/TV (%)	25.20 (5.91)	24.44 (6.15)	-0.76 (-1.03, -0.50)**	23.17 (4.12)	22.79 (4.28)	-0.39 (-0.65, -0.13)**	0.044*
Tb.N (mm^-1^)	1,41 (0.18)	1.38 (0.18)	-0.03 (-0.05, -0.01)**	1.32 (0.17)	1.33 (0.18)	0.01 (-0.01, 0.03)	0.006**
Tb.Th (mm)	0.25 (0.02)	0.25 (0.02)	0.00 (-0.00, -0.00)	0.24 (0.14)	0.25 (0.01)	0.00 (-0.00, 0.00)	0.815
Tb.Sp (mm)	0.67 (0.10)	0.69 (0.13)	0.02 (0.01, 0.04)**	0.73 (0.13)	0.73 (0.14)	0.00 (-0.00, 0.00)	0.007**
Ct.Th (mm)	1.14 (0.23)	1.04 (0.23)	-0.09 (-0.11, -0.08)**	1.04 (0.19)	0.99 (0.19)	-0.06 (-0.07, -0.04)**	0.003**
Ct.Po (%)	1.03 (0.55)	1.29 (0.63)	0.26 (0.13, 0.39)**	1.16 (0.64)	1.19 (0.58)	0.04 (-0.12, 0.19)	0.035*
*Bone strength*							
Stiffness (kN/mm)	81336.89 (22068.31)	72457.11 (21291.85)	-8879.74 (-10440.31, -7319.18)**	73570.957 (14710.304)	66303.29 (13779.24)	-7267.66 (-9536.70, -4998.62)**	0.235
FL (kN)	4378.82 (1180.15)	3879.54 (1117.73)	-499.28 (-582.68, -415.88)**	3981.055 (796.457)	3572.72 (754.31)	-408.34 (-530.21, -286.46)**	0.211

	ZOL− (N=67)			ZOL− (N=35)			p-value
	Mean T0 (SD)	Mean T2 (SD)	Δ T0-T2	Mean T0 (SD)	Mean T2 (SD)	Δ T0-T2	Δ ZOL- vs Δ ZOL+
Distal tibia							
*Bone area*							
Tb.Ar (mm^2^)*	748.13 (121.62)	762.99 (124.50)	14.87 (11.87, 17.86)**	765.92 (180.34)	776.15 (180.23)	10.23 (6.51, 13.94)**	0.057
Ct.Ar (mm^2^)*	153.78 (25.48)	138.52 (27.87)	-15.26 (-18.04, -12.49)**	141.64 (22.32)	131.46 (24.87)	-10.18 (-13.93, -6.43)**	0.029*
*Volumetric BMD*							
Tt.BMD (mg HA/cm^3^)*	298.47 (45.28)	276.86 (44.71)	-21.61 (-24.16, -19.07)**	269.85 (47.43)	256.44 (46.08)	-13.40 (-16.57, -10.24)**	0.000**
Tb.BMD (mg HA/cm^3^)*	182.88 (29.11)	181.21 (28.55)	-1.67 (-2.72, -0.63)**	163.22 (29.97)	164.20 (28.79)	0.98 (-0.09, 2.05)	0.001**
Ct.BMD (mg HA/cm^3^)*	854.13 (56.43)	793.22 (68.98)	-60.91 (-67.54, -54.28)**	821.95 (62.74)	778.48 (63.93)	-43.47 (-52.95, -33.99)**	0.002**
*Microarchitecture*							
Tb.BV/TV (%)*	26.80 (3.92)	26.76 (3.86)	-0.04 (-0.19, 0.12)	24.24 (3.96)	24.47 (3.86)	0.23 (0.04, 0.41)**	0.034*
Tb.N (mm^-1^)*	1.24 (0.17)	1.26 (0.19)	0.02 (0.00, 0.04)*	1.14 (0.18)	1.16 (0.22)	0.02 (0.04, 0.41)*	0.868
TbTh (mm)*	0.27 (0.02)	0.28 (0.02)	0.00 (-0.00, 0.00)	0.27 (0.03)	0.28 (0.02)	0.00 (0.00, 0.00)*	0.238
Tb.Sp (mm) *	0.77 (0.13)	0.77 (0.13)	-0.01 (-0.01, 0.00)	0.88 (0.25)	0.87 (0.28)	-0.00 (-0.02, 0.01)	0.759
Ct.Th (mm) *	1.57 (0.29)	1.44 (0.30)	-0.13 (-0.15, -0.11)**	1.46 (0.29)	1.38 (0.29)	-0.08 (-0.11, -0.05)**	0.007**
Ct.Po (%)*	3.18 (1.20)	3.77 (1.14)	0.60 (0.34, 0.85)**	3.30 (1.12)	3.66 (1.30)	0.37 (0.08, 0.65)**	0.246
*Bone strength*							
Stiffness (kN/mm)*	213100.71 (39481.86)	195705.76 (37301.11)	-17394.95 (-20298.84, -14491.05)**	187589.89 (35867.13)	175869.57 (33928.59)	-11720.32 (-15626.88, -7813.75)**	0.020*
FL (kN)*	11357.87 (1994.15)	10446.33 (1905.66)	-911.55 (-1053.97, -769,12)**	10042.40 (1830.25)	9410.36 (1746.88)	-632.04 (-829.63, -434.46)**	0.021*

* Significant delta (Δ) T0-T2 (p<0.05), ** significant (p<0.05) after correction for multiple testing (Benjamini-Hochberg), T0= HR-pQCT at initiation of androgen deprivation therapy T2= HR-pQCT at 24 months of androgen deprivation therapy, HR-pQCT; High Resolution peripheral quantitative computed tomography; Tb.Ar, trabecular bone area; Ct.Ar, cortical bone area; Tt.BMD, total bone mineral density; Tb.BMD, trabecular bone mineral density; Ct.BMD, cortical bone mineral density; Tb. BV/TV, trabecular bone volume fraction; Tb.N, trabecular number; Tb.Th, trabecular thickness; Tb.Sp, trabecular separation; Ct.Th, cortical thickness; Ct.Po, cortical porosity; FL, Failure load. ADT, Androgen Deprivation Therapy; ZOL, zoledronic acid 5mg; SD, standard deviation.

In the ZOL+ group, a similar pattern was observed: Ct.Ar, Tt.BMD, Ct.BMD, Ct.Th, stiffness, and FL decreased significantly, and Tb.Ar and Ct.Po increased significantly at the radius and tibia, while the changes in trabecular microarchitectural parameters were less pronounced (**Table 4**, **Figure 3**).

**Figure 4:**
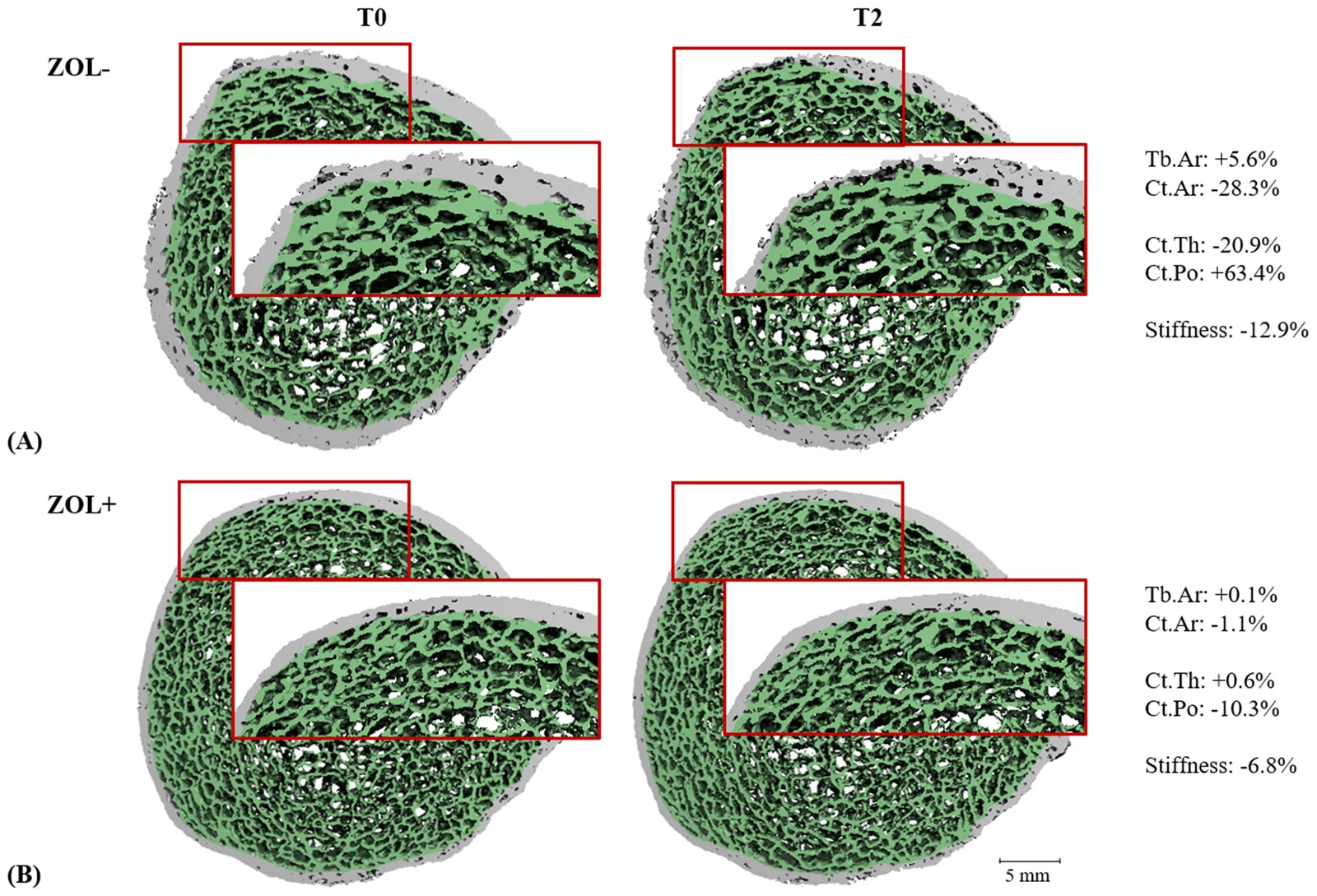
Three-dimensional reconstruction of HR-pQCT scans at the tibia, visualizing changes in cortical and trabecular bone area (Ct.Ar and Tb.Ar, resp.), cortical thickness (Ct.Th), cortical porosity (Ct.Po), and stiffness in two men with prostate cancer after two years androgen deprivation therapy. **(A)** Manwithout (ZOL-) and **(B)** man with (ZOL+) annual zoledronic acid.

When comparing the two groups, the HR-pQCT changes were significantly different for all parameters in the radius in favor of the ZOL+ group, except for Tb.Th, stiffness, and FL. (**Table 4**, **Supplementary Table 2**). Similar, although less pronounced, changes were observed at the distal tibia. An example of a three-dimensional visualization of HR-pQCT scans illustrating cortical changes at the distal tibia in the ZOL- and ZOL+ group is shown in **Figure 4**.

The proportion of men with a decrease ≥ LSC in Tt.BMD, Tb.BMD, and Ct.BMD ranged between 7.5% and 95.5% in the ZOL− group and between 0.0% and 90.0% in the ZOL+ group. The proportion of men with a decrease ≥ LSC in Tt.BMD was significantly higher in the ZOL− group than in the ZOL+ group and did not differ significantly for Tb.BMD and Ct.BMD (**Table 3**).

For completeness of the data, we presented the HR-pQCT results at T1 in **Supplementary Table 4**.

### 3.4 Fractures

In the ZOL− group, two men sustained a hip fracture and two a distal radius fracture. In the ZOL+ group, one man sustained a hip fracture, one a distal radius fracture, and one a clavicular fracture.

## 4 Discussion

In this observational study, we examined the two-year changes in central and peripheral aBMD assessed with DXA and in volumetric BMD, bone microarchitecture, and strength assessed with HR-pQCT after ADT initiation in men with PCa, who were or were not treated with annual zoledronic acid according to Dutch guidelines. After two years of ADT, we observed a significant decrease in aBMD at all sites in the ZOL− group. In the ZOL+ group, there was a significant increase in aBMD at the lumbar spine and a significant decrease in aBMD at the right leg. Using HR-pQCT, we observed a significant decrease in Ct.Ar, Tt.BMD, Ct.BMD, Ct.Th, stiffness, and failure load and a significant increase in Tb.Ar and Ct.Po at the radius and tibia in both the ZOL− and ZOL+ group.

These findings indicate that after the initiation of ADT in men with PCa, the loss of central and peripheral aBMD is accompanied by a significant loss in volumetric BMD in the cortical and trabecular compartments of the peripheral skeleton, an increase in trabecular area, a decrease in cortical area, and a corresponding decrease in bone strength. While treatment with ZOL preserved aBMD (and increased lumbar spine aBMD), HR-pQCT analyses revealed deterioration of total, trabecular, and cortical volumetric BMD, cortical area and thickness, and bone strength at the peripheral skeleton.

We observed that 24 months of ADT resulted in a significant decrease in central and peripheral aBMD in the ZOL− group. Consistent with 12-month findings in several prior studies, we found a comparable 4% decrease in aBMD at the LS, TH and FN in the ZOL− group (11, 13, 39–41). A systematic review and meta-analysis of 533 PCa patients with and without ADT (6–36 months) also showed a significant decline in aBMD at the LS, FN, and TH regions of PCa patients treated with ADT compared to controls (42). In a previous study of 62 men followed for 24 months, aBMD at the LS, FN, and TH declined most during the first year of ADT and continued to decrease more modestly and non-significantly in the second year (43). A secondary analysis of a prospective cohort study in 160 men with PCa (80 ADT users and 80 controls) aged 50+, showed that ADT resulted in a significant LS aBMD decline at 12 months, while subsequent changes over years 2–3 were modest and not significantly different from controls (44). This suggests that a substantial proportion of aBMD loss occurs during the first year following ADT initiation, after which the rate of bone loss becomes less pronounced. This decrease in aBMD after ADT initiation is more pronounced than bone loss typically observed in aging men (45).

In addition to the notable reduction in aBMD in LS, FN and TH in the ZOL− group, we observed a decrease in aBMD in the whole body, arms, and legs. Data on peripheral aBMD in this population are scarce. One study investigating the long-term effects of ADT on aBMD, measured using a portable DXA at ultra-distal region of the forearm, showed that 35% of patients with an initial T-score between −1.0 and −2.5, had a T-score <-2.5 after one year of ADT, which increased to 60% after two years of ADT (46). These results, together with ours, emphasize that both central and peripheral aBMD decrease following the start of ADT. In the ZOL+ group, we observed a significant increase in aBMD at the LS, and no significant changes in FN, TH, arms and left leg. These findings are in line with an RCT in 44 men with PCa with a normal aBMD, in which a single ZOL administration at the start of ADT significantly improved LS aBMD at 12 and 24 months compared with delayed administration, while TH and FN aBMD did not change (47). A previous placebo-controlled RCT by Cheung et al. (22) in 76 men administering a single dose of ZOL 6 weeks after ADT initiation, showed an 8.4% higher aBMD at the LS, 4.4% at TH, and 3.7% at the total radius after 24 months. However, the effect of ZOL on aBMD at the ultra-distal radius was not significant. These findings suggest that the effect of ZOL on aBMD during ADT is dose- and site-dependent, and that early and repeated ZOL dosing may be necessary for optimal site-specific preservation during ADT. However, available literature is heterogeneous in baseline aBMD, fracture risk, and the timing and frequency of ZOL doses, limiting the ability to draw firm conclusions.

Most studies on the effects of ADT on bone have assessed aBMD using DXA, while only a few have combined these measurements with HR-pQCT. In this study, we observed a significant decrease in volumetric BMD, cortical area and thickness, and bone strength and an increase in trabecular area at the radius and tibia in both the ZOL− and ZOL+ group. The changes in area suggest endosteal trabecularization (i.e., cortical bone thinning at the endosteal boundary).

The decline in BMD at the radius and tibia after 24 months was more pronounced in our ZOL− group than in our ZOL+ group. This aligns with prior 12-month studies that examined populations comparable to our ZOL− group. An observational study by Hamilton et al. (19), in men on ADT without AOM, reported that ADT resulted in loss of both cortical and trabecular bone. A more recent study by Handforth et al. (13) also reported a decrease in total and cortical BMD, as well as in cortical area at the distal radius in their ADT group (without AOM) compared to healthy age-matched controls. In their follow-up study, they also reported a significant reduction in total and trabecular BMD and mechanical properties at the vertebrae based on QCT (20). In both studies, first−generation HR−pQCT scanners were used, which have a lower spatial resolution than second−generation devices. Consequently, not all trabecular microarchitecture parameters can be measured directly with this generation systems, in contrast to with the second−generation HR−pQCT system used in this work. Together with our longer follow-up period than in these studies, our observations support ADT−induced skeletal deterioration.

In the ZOL+ group, the deterioration in BMD, cortical bone microarchitectural parameters, and failure load at the radius and tibia contrasted with the unchanged aBMD at most central and peripheral sites. This is in line with the two-year RCT by Cheung et al. (22) who reported a decrease in total, cortical and trabecular BMD at the tibia, and a modest increase of total BMD at the radius after a single dose of ZOL, compared with placebo, despite the significant treatment effect on aBMD at all sites except the ultra-distal radius. This apparent discrepancy, of stable or improving central and peripheral DXA-derived aBMD and deteriorated peripheral microarchitecture with ZOL treatment, underscores the additional value of complementary assessments. It furthermore suggests that the unbalanced bone remodeling with increased resorption relative to formation induced by ADT may persist despite treatment with ZOL and cause subtle microarchitectural deterioration that is detectable with HR-pQCT but not captured by DXA-derived aBMD.

Our study has several strengths and limitations. The strengths are the use of real-world data, 24-month follow-up, and dual-modality bone assessment. Real-world data are important for understanding the performance of fracture prevention strategies outside controlled research settings and reflect the existing complexity of routine clinical care. Our findings contribute to this understanding by demonstrating clinically relevant skeletal deterioration in a representative population of men with prostate cancer undergoing ADT. Additionally, our 24−month follow-up provides a stronger evaluation of sustained effects on bone microarchitecture than most previous HR−pQCT studies that assessed changes with ADT over only 12 months. Besides DXA, we included HR-pQCT, which, together with DXA and fracture data, provides a more complete impression of the effects of ADT with and without ZOL on bone. Other clinically relevant predictors of microarchitectural deterioration and bone loss were not evaluated in the present cohort and represent an important direction for future research in larger populations. The use of real-world data is also a limitation of our study given its susceptibility to selection bias as treatment decisions for both ADT and ZOL are influenced by clinician judgment, patient-related profiles, and preferences. However, the indication for treatment was based on guideline-recommended fracture risk criteria, reflecting real-world clinical decision-making. In addition, secondary causes of bone fragility (such as primary hyperparathyroidism, vitamin D deficiency, CKD, COPD and use of glucocorticoids) were found in 20 men in the ZOL- group and 18 men in the ZOL+ group). A sensitivity analysis excluding these patients (data not shown) however showed comparable results to those of the primary analysis, and the overall conclusions remained unchanged. Second, DXA-based aBMD at the arms and legs is based on a different scan region than the HR-pQCT scan regions, so peripheral DXA and HR-pQCT measurements at the distal radius and tibia are not directly comparable. Similarly, microarchitectural and strength effects at central sites cannot be assessed using DXA nor HR-pQCT and thus require separate study. In that regard, Gibson et al. recently found a reduction in mechanical properties of vertebrae after twelve months of ADT using QCT-based FE-modeling (20). Third, alternative material properties or loading conditions could have been used in the µFE-analysis. However, the approach we used (including a uniform Young’s Modulus and axial compression), is standardly used in HR-pQCT based FE analyses and has been extensively validated experimentally (29, 32, 48, 49). Finally, no literature-based LSCs were identified for total-body and arm/leg aBMD, consequently a LSC of 4.5% as established for TH and FN was also applied at these sites (24, 26, 36). Despite these limitations, the results of our study may provide a basis for future site-specific investigations to improve the combined interpretation of aBMD and HR−pQCT data.

### 4.1 Conclusion

This study showed that 24 months of ADT leads to significant deterioration of central and peripheral aBMD and of peripheral bone microarchitecture and strength in men with prostate cancer. Although treatment with zoledronic acid preserved aBMD and mitigated microarchitectural decline, loss of peripheral cortical bone structure and bone strength was observed. These findings emphasize the importance of early risk assessment and guideline-based intervention and highlight the need for continued improvement of bone health strategies in men with prostate cancer undergoing ADT.

## Data Availability

The original contributions presented in the study are included in the article/**Supplementary Material**. Further inquiries can be directed to the corresponding author.
