# Supplementary material for: Central and peripheral areal BMD, bone microarchitecture, and strength after initiation of androgen deprivation therapy in men with prostate cancer: a two-year follow-up study
Source: Front Endocrinol (Lausanne). 2026 Aug 13;17:1862001. doi: 10.3389/fendo.2026.1862001 (PMC13519974; doi:10.3389/fendo.2026.1862001)
Supplement: Supplementary file 1 [file Table1.pdf]

Supplemental Table 1: Percentage of change in Areal BMD (g/cm²) measured by DXA in men with prostate cancer after two years androgen deprivation therapy, with and without annual zoledronic acid

	ZOL− (N=67)	ZOL+ (N=40)	Difference in absolute percentage between ZOL+ and ZOL−
	**Mean change (%)**	**Mean change (%)**	**∆ ZOL+ minus ZOL−**
Lumbar Spine aBMD (g/cm2)	-3.2 (-4.4, -1.9)**	3.6 (2.3, 5.0)**	-6.8 (-8.7, -4.9)**
Femoral Neck aBMD (g/cm2)	-5.0 (-6.2, -3.7)**	-0.2 (-1.6, -1.2)	-4.7 (-6.7, -2.8)**
Total Hip aBMD (g/cm2)	-4.1 (-5.2, -3.0)**	1.6 (0.1, 3.2)*	-5.7 (-7.6, -3.9) **
Total Body aBMD (g/cm2)	-4.5 (-5.4, -3.5)**	-1.0 (-2.2, 0.2)	-3.4 (-4.9,-2.0)**
Left Arm aBMD (g/cm2)	-4.7 (-6.1, -3.2)**	-1.2 (-2.5, 0.1)	-3.4 (-5.6, -1.3)**
Right Arm aBMD (g/cm2)	-4.8 (-6.1, -3.4)**	-1.2 (-2.2, -0.2)*	-3.6 (-5.5, -1.7)**
Left Leg aBMD (g/cm2)	-3.0 (-4.7, -1.1)**	1.6 (-2.7, 5.8)	-4.5 (-8.4, -0.6)**
Right Leg aBMD (g/cm2)	-4.1 (-5.6, -2.6)**	-2.3 (-3.4, -1.2)**	-1.8 (-4.0, 0.4)

* significant delta (∆) T0-T2 ($p<0.05$) ** significant after correction for multiple testing (Benjamini-Hochberg), BMD; bone mineral density, DXA; Dual-energy X-ray absorptiometry, ADT, ZOL; zoledronic acid 5mg, SD; standard deviation
