# Supplementary material for: Central and peripheral areal BMD, bone microarchitecture, and strength after initiation of androgen deprivation therapy in men with prostate cancer: a two-year follow-up study
Source: Front Endocrinol (Lausanne). 2026 Aug 13;17:1862001. doi: 10.3389/fendo.2026.1862001 (PMC13519974; doi:10.3389/fendo.2026.1862001)
Supplement: Supplementary file 2 [file Table2.pdf]

Supplemental Table 2: Longitudinal relative (%) changes in bone area, volumetric bone mineral density and microarchitecture Measured by HR-pQCT in men with prostate cancer after two years androgen deprivation therapy, with and without annual zoledronic acid

	ZOL− (N=67)	ZOL+ (N=35)	% difference between ZOL+ and ZOL−
	**Mean change (%)**	**Mean change (%)**	**∆ ZOL+ minus ZOL−**
**Distal Radius**			
Bone area			
Tb.Ar (mm^2)	2.6 (2.2, 3.1)**	1.4 (1.0, 1.9)**	1.2 (0.5, 1.8)**
Ct.Ar (mm^2)	-10.1 (-11.5, -8.7)**	-6.5 (-8.2, -4.7)**	-3.6 (-5.9, -1.3)**
Volumetric BMD			
Tt.BMD (mg HA/cm^3)	-8.8 (-9.9, -7.8)**	-5.5 (-6.8, -4.3)**	-3.3 (-5.0, -1.6)**
Tb.BMD (mg HA/cm^3)	-4.1 (-5.2, -2.9)**	-1.9 (-2.8,-1.1)**	-2.1 (-3.5,-0.7)**
Ct.BMD (mg HA/cm^3)	-5.2 (-6.0, -4.5)**	-3.7 (-4.5, -2.9)**	-1.5 (-2.7, -0.4)**
Microarchitecture			
Tb.BV/TV (%)	-3.3 (-4.5 ,-2.1)**	-1.7 (-2.8, -0.6)**	-1.6 (-3.2, -0.1)*
Tb.N (mm^{-1})	-1.8 (-3.3, -0.2)*	0.9 (-0.4, 2.2)	-2.7 (-5.0, -0.4)*
Tb.Th (mm)	0.3 (-0.4, 1.0)	0.5 (-0.1, 1.2)	-0.2 (-1.3, 0.9)*
Tb.Sp (mm)	3.2 (1.6, 4.8)**	0.3 (-0.5, 1.1)	2.9 (1.1, 4.7)**
Ct.Th (mm)	-8.4 (-9.8, -7.1)**	-5.5 (-7.0, -4.0)**	-2.9 (-5.1, -0.7)**
Ct.Po (%)	42.3 (27.1, 57.4)**	13.9 (-2.1, 30.0)	28.3 (4.6, 52.1)*
Bone strength			
Stiffness (kN/mm)	-11.2 (-13.2, -9.2)**	-9.8 (-12.6, -7.0)**	-1.4 (-4.8, 1.9)
FL (kN)	-11.6 (-13.5, -9.6)**	-10.2 (-13.0, -7.4)**	-1.4 (-4.7, 1.9)

	ZOL− (N=67)	ZOL+ (N=40)	% difference between ZOL+ and ZOL−
	Δ T0-T2 (%)	Δ T0-T2 (%)	Δ ZOL+ minus ZOL−
**Distal tibia**			
Bone area			
Tb.Ar (mm^2)*	2.0 (1.6, 2.4)**	1.4 (0.9, 1.9)**	0.6 (-0.1, 1.2)
Ct.Ar (mm^2)*	-10.1 (-12.1, -8.2)**	-7.3 (-9.9, -4.6)**	-2.9 (-6.1, 0.4)
Volumetric BMD			
Tt.BMD (mg HA/cm^3)*	-7.3 (-8.2, -6.4)**	-5.0 (-6.3, -3.7)**	-2.3 (-3.9, -0.8)**
Tb.BMD (mg HA/cm^3)*	-0.9 (-1.4, -0.3)**	0.8 (-0.0, 1.7)	-1.7 (-2.7, -0.7)**
Ct.BMD (mg HA/cm^3)*	-7.2 (-8.1, -6.4)**	-5.3 (-6.4,-4.1)**	-2.0 (-3.4, -0.6)*
Microarchitecture			
Tb.BV/TV (%)*	-0.1 (-0.7, 0.5)	1.1 (0.2, 2.0)**	-1.2 (-2.2, -0.1)*
Tb.N (mm^{-1})*	1.6 (0.3, 2.9)**	1.6 (-0.1, 3.3)	-0.0 (-2.1, 2.1)
TbTh (mm)*	0.5 (-0.0, 1.0)	1.0 (0.2, 1.9)**	-0.6 (-1.5, 0.3)
Tb.Sp (mm) *	-0.7 (-1.6, 0.2)	-0.8 (-2.0, 0.4)	0.2 (-1.3, 1.6)
Ct.Th (mm) *	-8.4 (-9.9, -6.9)**	-5.4 (-7.4,-3.4)**	-3.0 (-5.5, -0.5)*
Ct.Po (%)*	25.1 (14.7, 35.6)**	14.2 (3.4, 25.1)**	10.9 (-4.8, 26.6)
Bone strength			
Stiffness (kN/mm)*	-8.1 (-9.4, -6.7)**	-6.1 (-8.1 ,-4.1)**	-2.0 (-4.3, 0.3)
FL (kN)*	-8.0 (-9.3, -6.7)**	-6.2 (-8.1, -4.2)**	-1.8 (-4.0, 0.4)

* Significant delta (Δ) T0-T2 ($p<0.05$), ** significant ($p<0.05$) after correction for multiple testing (Benjamini-Hochberg), T0= HR-pQCT at initiation of androgen deprivation therapy T2= HR-pQCT at 24 months of androgen deprivation therapy, HR-pQCT; High Resolution peripheral quantitative computed tomography, Tb.Ar, trabecular bone area; Ct.Ar, cortical bone area; Tt.BMD, total bone mineral density; Tb.BMD, trabecular bone mineral density; Ct.BMD, cortical bone mineral density; Tb. BV/TV, trabecular bone volume fraction; Tb.N, trabecular number; Tb.Th, trabecular thickness; Tb.Sp, trabecular separation; Ct.Th, cortical thickness; Ct.Po, cortical porosity; FL, Failure load. ADT; Androgen Deprivation Therapy, ZOL; zoledronic acid 5mg, SD; standard deviation
