# Supplementary material for: Central and peripheral areal BMD, bone microarchitecture, and strength after initiation of androgen deprivation therapy in men with prostate cancer: a two-year follow-up study
Source: Front Endocrinol (Lausanne). 2026 Aug 13;17:1862001. doi: 10.3389/fendo.2026.1862001 (PMC13519974; doi:10.3389/fendo.2026.1862001)
Supplement: Supplementary file 3 [file Table3.pdf]

Supplemental Table 3: Longitudinal absolute changes in aBMD (g/cm²) in men with prostate cancer after one year androgen deprivation therapy, with and without annual zoledronic acid

	ZOL− (N=67)			ZOL + (N = 40)			p-value (absolute)
	**Mean T0 (SD)**	**Mean T1 (SD)**	**Mean difference T0 – T1**	**Mean T0 (SD)**	**Mean T1 (SD)**	**Mean difference T0 – T1**	**∆ ZOL- vs ∆ ZOL+**
Lumbar Spine aBMD (g/cm²)	1.139 (0.160)	1.110 (0.166)	-0.029 (-0.040. -0.018)*	1.017 (0.216)	1.045 (0.216)	0.029 (0.018. 0.039)*	0.000*
Femoral Neck aBMD (g/cm²)	0.816 (0.106)	0.792 (0.108)	-0.024 (-0.031. -0.016)*	0.707 (0.117)	0.714 (0.119)	0.007 (-0.001. 0.014)	0.000*
Total Hip aBMD (g/cm²)	1.009 (0.117)	0.980 (0.126)	-0.029 (-0.041. -0.018)*	0.892 (0.122)	0.898 (0.125)	0.006 (-0.006. 0.019)	0.000*
Total Body aBMD (g/cm²)	1.197 (0.120)	1.167 (0.122)	-0.030 (-0.038. -0.023)*	1.115 (0.117)	1.103 (0.115)	-0.012 (-0.023. -0.001)*	0.006*
Left Arm aBMD (g/cm²)	0.865 (0.069)	0.841 (0.076)	-0.024 (-0.034. -0.015)*	0.810 (0.062)	0.806 (0.070)	-0.004 (-0.018. 0.010)	0.015*
Right Arm aBMD (g/cm²)	0.830 (0.072)	0.851 (0.081)	-0.023 (-0.034. -0.012)*	0.819 (0.066)	0.814 (0.067)	-0.005 (-0.016. 0.006)	0.040*
Left Leg aBMD (g/cm²)	1.297 (0.196)	1.270 (0.205)	-0.027 (-0.039. -0.015)*	1.195 (0.159)	1.198 (0.156)	0.003 (-0.030. 0.035)	0.046*
Right Leg aBMD (g/cm²)	1.295 (0.183)	1.270 (0.193)	-0.025 (-0.040. -0.010)*	1.234 (0.224)	1.226 (0.205)	-0.008 (-0.025. 0.008)	0.157

* significant $p<0.05$, Mean difference T0 – T1: not corrected for multiple testing, T0= aBMD measured at initiation of androgen deprivation therapy, T1= at 12 months of androgen deprivation therapy, T2= aBMD measured at 24 months of androgen deprivation therapy, BMD; bone mineral density, DXA; Dual-energy X-ray absorptiometry, ADT; Androgen Deprivation Therapy, ZOL; zoledronic acid 5mg, SD; standard deviation
