# Supplementary material for: Central and peripheral areal BMD, bone microarchitecture, and strength after initiation of androgen deprivation therapy in men with prostate cancer: a two-year follow-up study
Source: Front Endocrinol (Lausanne). 2026 Aug 13;17:1862001. doi: 10.3389/fendo.2026.1862001 (PMC13519974; doi:10.3389/fendo.2026.1862001)
Supplement: Supplementary file 4 [file Table4.pdf]

Supplemental Table 4: Longitudinal absolute changes in bone area, volumetric bone mineral density and microarchitecture measured by HR-pQCT in men with prostate cancer after one year androgen deprivation therapy, with and without annual zoledronic acid

	ZOL− (N=67)			ZOL+ (N = 35)			p-value (absolute)
	**Mean T0 (SD)**	**Mean T1 (SD)**	**Mean difference T0 – T1**	**Mean T0 (SD)**	**Mean T1 (SD)**	**Mean difference T0 – T1**	**∆ ZOL- vs ∆ ZOL+**
**Distal Radius**							
Bone area							
Tb.Ar (mm^2)	305.10 (61.78)	306.455 (60.059)	3.159 (2.286. 4.032)*	310.32 (65.11)	312.840 (65.719)	2.523 (1.469, 3.577)*	0.002*
Ct.Ar (mm^2)	78.64 (16.63)	75.442 (17.112)	-3.261 (-4.117. -2.405)*	72.09 (11.96)	69.483 (11.744)	-2.606 (-3.599, -1.612)*	0.001*
Volumetric BMD							
Tt.BMD (mg HA/cm^3)	320.51 (66.80)	308.606 (67.367)	-12.456 (-14.701. -10.212)*	294.61 (46.99)	284.826 (45.850)	-9.786 (-12.826, -6.745)*	0.000*
Tb.BMD (mg HA/cm^3)	178.79 (38.94)	176.453 (40.310)	-2.242 (-3.034. -1.451)*	163.17 (29.97)	161.231 (29.717)	-1.940 (-3.042, -0.838)*	0.002*
Ct.BMD (mg HA/cm^3)	864.58 (53.85)	841.126 (59.356)	-23.395 (-27.667. -19.124)*	853.45 (53.47)	833.680 (57.578)	-19.766 (-24.907, -14.625)*	0.004*
Microarchitecture							
Tb.BV/TV (%)	25.20 (5.91)	24.983 (6.067)	-0.205 (-0.354. -0.055)*	23.17 (4.12)	23.003 (4.235)	-0.171 (-0.377, 0.034)	0.044*
Tb.N (mm^{-1})	1.41 (0.18)	1.399 (0.180)	-0.007 (-0.027. 0.012)	1.32 (0.17)	1.325 (0.185)	0.009 (-0.011, 0.030)	0.006*
Tb.Th (mm)	0.25 (0.02)	0.249 (0.021)	0.000 (-0.002. 0.001)	0.24 (0.14)	0.245 (0.013)	0.000 (-0.001, 0.002)	0.815
Tb.Sp (mm)	0.67 (0.10)	0.680 (0.112)	0.008 (0.001. 0.015)*	0.73 (0.13)	0.728 (0.129)	0.000 (-0.006, 0.007)	0.007*
Ct.Th (mm)	1.14 (0.23)	1.102 (0.237)	-0.036 (-0.048. -0.026)*	1.04 (0.19)	1.011 (0.189)	-0.031 (-0.043, -0.019)*	0.003*
Ct.Po (%)	1.03 (0.55)	1.124 (0.573)	0.108 (0.001. 0.215)*	1.16 (0.64)	1.171 (0.572)	0.014 (-0.088, 0.117)	0.035*
Bone strength							
Stiffness (kN/mm)	81336.89 (22068.31)	77437.303 (22357.683)	-3589.732 (-5396.391. -1783.073)*	73570.957 (14710.304)	69500.994 (12765.988)	-4069.963 (-5918.065, -2221.861)*	0.235
FL (kN)	4378.82 (1180.15)	4118.267 (1148.885)	-244.289 (-314.812. -173.765)*	3981.055 (796.457)	3745.958 (702.254)	-235.097 (-332.787, -137.408)*	0.211

	ZOL− (N=67)			ZOL+ (N = 35)			
	**Mean T0 (SD)**	**Mean T1 (SD)**	**Mean difference T0 – T1**	**Mean T0 (SD)**	**Mean T1 (SD)**	**Mean difference T0 – T1**	**∆ ZOL- vs ∆ ZOL+**
**Distal Tibia**							
Bone area							
Tb.Ar (mm^2)	748.13 (121.62)	753.397 (122.733)	7.565 (5.328, 9.802)*	765.92 (180.34)	767.584 (183.691)	7.747 (5.013, 10.482)*	0.919
Ct.Ar (mm^2)	153.78 (25.48)	145.914 (27.349)	-7.929 (-9.819, -6.038)*	141.64 (22.32)	134.424 (24.347)	-7.713 (-10.452, -4.974)*	0.894
Volumetric BMD							
Tt.BMD (mg HA/cm^3)	298.47 (45.28)	287.658 (45.747)	-10.552 (-12.185, -8.918)*	269.85 (47.43)	263.689 (46.132)	-9.287 (-11.358, -7.216)*	0.343
Tb.BMD (mg HA/cm^3)	182.88 (29.11)	182.518 (28.694)	0.297 (-0.223, 0.817)	163.22 (29.97)	165.895 (28.961)	0.953 (0.214, 1.691)*	0.140
Ct.BMD (mg HA/cm^3)	854.13 (56.43)	821.848 (65.317)	-32.135 (-36.566, -27.704)*	821.95 (62.74)	796.942 (63.278)	-28.484 (-35.631, -21.338)*	0.359
Microarchitecture							
Tb.BV/TV (%)	26.80 (3.92)	26.824 (3.865)	0.126 (0.045, 0.207)*	24.24 (3.96)	24.587 (3.818)	0.134 (-0.008, 0.276)	0.911
Tb.N (mm^{-1})	1.24 (0.17)	1.252 (0.169)	0.012 (-0.003, 0.027)	1.14 (0.18)	1.173 (0.220)	0.029 (0.004, 0.055)*	0.216
Tb.Th (mm)	0.27 (0.02)	0.276 (0.023)	0.001 (0.000, 0.003)*	0.27 (0.03)	0.276 (0.025)	0.003 (0.000, 0.005)*	0.422
Tb.Sp (mm)	0.77 (0.13)	0.770 (0.127)	-0.006 (-0.012, 0.000)	0.88 (0.25)	0.862 (0.270)	-0.012 (-0.021, -0.002)*	0.255
Ct.Th (mm)	1.57 (0.29)	1.505 (0.295)	-0.063 (-0.078, -0.049)*	1.46 (0.29)	1.415 (0.289)	-0.059 (-0.076, -0.042)*	0.712
Ct.Po (%)	3.18 (1.20)	3.294 (1.265)	0.118 (-0.061, 0.297)	3.30 (1.12)	3.358 (1.181)	0.058 (-0.204, 0.320)	0.695
Bone strength							
Stiffness (kN/mm)	213100.71 (39481.86)	203478.303 (38648.316)	-8687.420 (-10621.116, -6753.723)*	187589.89 (35867.13)	179363.547 (35422.811)	-10136.276 (-14132.074, -6140.479)*	0.659
FL (kN)	11357.87 (1994.15)	10840.978 (1962.948)	-469.449 (-562.978, -375.920)*	10042.40 (1830.25)	9599.775 (1819.890)	-538.534 (-738.599, -338.469)*	0.632

* significant $p<0.05$, Mean difference T0 – T1: not corrected for multiple testing, T0= HR-pQCT at initiation of androgen deprivation therapy, T1= at 12 months of androgen deprivation therapy, T2= HR-pQCT at 24 months of androgen deprivation therapy, HR-pQCT; High Resolution peripheral quantitative computed tomography, Tb.Ar, trabecular bone area; Ct.Ar, cortical bone area; Tt.BMD, total bone mineral density; Tb.BMD, trabecular bone mineral density; Ct.BMD, cortical bone mineral density; Tb. BV/TV, trabecular bone volume fraction; Tb.N, trabecular number; Tb.Th, trabecular thickness; Tb.Sp, trabecular separation; Ct.Th, cortical thickness; Ct.Po, cortical porosity; FL, Failure load. ADT; Androgen Deprivation Therapy, ZOL; zoledronic acid 5mg, SD; standard deviation
